# Identification of a high-risk immunogenic prostate cancer patient subset as candidates for T-cell engager immunotherapy and the introduction of a novel albumin-fused anti-CD3 × anti-PSMA bispecific design

**DOI:** 10.1038/s41416-022-01994-1

**Published:** 2022-10-15

**Authors:** Eske N. Glud, Martin Rasmussen, Yonghui Zhang, Ole A. Mandrup, Paul Vinu Salachan, Michael Borre, Karina Dalsgaard Sørensen, Kenneth A. Howard

**Affiliations:** 1 Interdisciplinary Nanoscience Center (iNANO), Department of Molecular Biology and Genetics, Aarhus University, 8000 Aarhus, Denmark; 2 Department of Molecular Medicine, Aarhus University Hospital & Department of Clinical Medicine, Aarhus University, 8200 Aarhus, Denmark; 3 Department of Urology, Aarhus University Hospital & Department of Clinical Medicine, Aarhus University, 8200 Aarhus, Denmark

**Keywords:** Prostate cancer, Prognostic markers, Immunotherapy

## Abstract

**Background:**

Cancer immunotherapies such as bispecific T-cell engagers have seen limited adoption in prostate cancer (PC), possibly due to differing levels of cancer receptor expression and effector T-cell infiltration between patients and inherent defects in T-cell engager design.

**Methods:**

CD8^+^ T-cell infiltration and PSMA expression were determined by RNA sequencing of primary PC tissue samples from 126 patients with localised PC and 17 patients with metastatic PC. Prognostic value was assessed through clinical parameters, including CAPRA-S risk score. A panel of albumin-fused anti-CD3 ×  anti-PSMA T-cell engagers with different neonatal Fc receptor (FcRn) affinity were characterised by flow cytometry, Bio-Layer Interferometry and functional cellular assays.

**Results:**

A subset of patients with localised (30/126 = 24%) and metastatic (10/17 = 59%) PC showed both high PSMA expression and high CD8^+^ T-cell enrichment. The High/High phenotype in localised PC associated with a clinically high-risk cancer subtype, confirmed in an external patient cohort (*n* = 550, PRAD/TCGA). The T-cell engagers exhibited tunable FcRn-driven cellular recycling, CD3 and PSMA cellular engagement, T-cell activation and PSMA level-dependent cellular cytotoxicity.

**Conclusion:**

This work presents an albumin-fused bispecific T-cell engager with programmable FcRn engagement and identifies a high-risk PC patient subset as candidates for treatment with the T-cell engager class of immuno-oncology biologics.

## Background

Multiple cancers have seen long-term death rate declines; however, prostate cancer (PC) remains the second leading cause of cancer death in men in Western countries [1, 2]. Curatively intended treatments are offered with localised disease, but recurrence occurs in ~30% of the patients, who, therefore, require additional therapeutic intervention [3, 4]. Palliative treatments are available such as androgen deprivation therapy (ADT) for metastatic PC, however, resistance eventually develops, and cancer becomes classified as metastatic castration-resistant prostate cancer (mCRPC) with very poor prognosis [5]. Immunotherapy has yet to be successfully adopted for PC, with immune checkpoint inhibition failing to improve overall survival in clinical trials of unselected patient populations, and the Sipuleucel-T cancer vaccine failing to widely impact clinical practice due to modest benefit and high cost [6–8]. This likely reflects different cancer evasion mechanisms and immune environments between patients [9]. Bispecific T-cell engagers provide a conduit between the patient’s cytotoxic CD8^+^ T-cells, commonly through CD3, and the tumour cells resulting in the formation of an immunological synapse and concomitant effector T-cell-mediated killing of the cancer cell [10]. Interaction between CD3 and tumour antigen is independent of the canonical MHC-TCR pathway, thus, overcoming immune evasion mechanisms such as MHC-1 downregulation [11]. Efficacy is dependent on tumour resident CD8^+^ T-cells, the ratio of effector to target cells, and raised levels of target antigen in malignant tissue [12–14]. Multiple bispecific T-cell engagers targeting a variety of antigens are under development, with Blinatumomab (Blincyto®) approved for the treatment of acute lymphoblastic leukaemia [15]. The design involves the removal of the fragment crystallisable (Fc) region to avoid adverse non-specific immune induction such as cytokine release syndrome (CRS) mediated by Fc interaction with Fcƴ receptor-bearing immune cells [16]. This, however, results in loss of Fc engagement and neonatal Fc receptor (FcRn)-driven cellular recycling in the vasculature endothelium resulting in a short plasma half-life and requirement for continual infusion into the patient [17]. The long circulatory half-life of human serum albumin (HSA) of ~19 days, is also facilitated by engagement with FcRn [18] but unlike IgGs does not react with Fcƴ receptors, and has been utilised as a safe half-life extension technology in marketed albumin-based drug designs [19, 20]. Single-point amino acid mutations in albumin domain III, lying within the main binding interface with FcRn, has been shown to alter binding affinity and modulate the circulatory half-life [21]. Our lab has recently engineered a panel of anti-CD3 × anti-EGFR bispecific T-cell engager-albumin genetic fusions engineered with different FcRn affinity for programming the half-life as a method to maximise efficacy [22].

Prostate-specific membrane antigen (PSMA, encoded by *FOLH1*) is upregulated in most prostate cancers and utilised as a therapeutic and diagnostic target [1, 23–25]. Several bispecific constructs targeting PSMA are currently under evaluation in Phase-1 clinical trials for PC. These include CC-1 [26], TNB-585 [27], CCW702 [28], and AMG 160 [29] with selection criteria not based on PSMA and effector T-cell levels, that likely results in the inclusion of patients with either immunologically “cold” or PSMA negative tumours. AMG 212 is an anti-CD3 × anti-PSMA scFv bispecific with promising Phase-1 clinical results, however, requirement of continuous infusion was necessary due to lack of Fc region and the project terminated [30]. AMG 160 is a continuation of the AMG 212 bispecific design with the addition of a silenced Fc region improving half-life to 6.1 days [31]. In a recent Phase-1 clinical trial, 27% of patients treated with AMG 160 had confirmed Prostate-specific antigen (PSA) response, yet overall, the PSA response remained heterogeneous with ~a third of patients being non-responders and 84.4% of patients experiencing CRS [29, 32]. This suggests that shortcomings remain and potentially could be overcome by modifying the bispecific T-cell engager design and improving the selection of patients included in clinical trials [33]. PSMA levels combined with CD8^+^ effector T-cell infiltration presently remains underexplored. Identifying PSMA/CD8^+^ T-cell correlation in PC tumours could lead to the personalised application of bispecific T-cell engagers in pre-selected patient subsets.

In this work, we have designed, produced, and characterised a panel of novel scFv anti-CD3 × human domain antibody anti-PSMA HSA-genetically fused bispecific T-cell engagers (termed AlproTox) engineered with either null-binding (NB), wild-type (WT) or high-binding (HB) FcRn affinity to potentially tune circulatory half-life and give long-lasting effects. Furthermore, we have identified a high-risk immunogenic PC patient subset as potential candidates for T-cell engager immunotherapy.

## Methods

### Patient samples

Total RNA sequencing was performed on 7 normal (N) prostate tissue samples, 31 adjacent-normal (AN) prostate tissue samples, 126 primary PC tissue samples (LPC) from patients who underwent curatively intended radical prostatectomy (RP) for clinically localised PC, and 17 primary tumour samples (MPC) from patients with metastatic PC who underwent palliative transurethral resection of the prostate (TURP). The normal prostate tissue samples were from patients who underwent cystoprostatectomy due to bladder cancer and were histopathologically confirmed to be free of PC. AN samples were from LPC patients, who underwent RP [34].

All samples were collected at Department of Urology, Aarhus University Hospital or Holstebro Hospital, as described previously [34]. Immediately following surgery, fresh prostate tissue biopsies were obtained and stored at −80 °C in TissueTek. For RNA extraction, TissueTek samples were cut in ~40 sections (20-µm thickness) and the first and last sections stained with haematoxylin and eosin (H&E) and evaluated by a trained pathologist to assess areas of malignant (PC) and benign (AN/N) prostate tissue. Total RNA was extracted from the remaining ~38 sections using the RNeasy Plus Mini Kit (Qiagen, Hilden, Germany), as described previously [35]. RNA concentration and quality was assessed using a NanoQuant and an Agilent 2100 Bioanalyzer (RIN ≥ 7), respectively.

### RNA sequencing and xCell analyses

Sequencing libraries were generated using either the ScriptSeq RNA-seq Library Kit with the Ribo-Zero Magnetic Gold Kit from Illumina (San Diego, California, USA) (N = 7; AN = 11; LPC = 52) or the KAPA RNA HyperPrep Kit with KAPA RiboErase Kit from Roche (Basel, Switzerland) (AN = 20; LPC = 74; MPC = 17). All libraries were sequenced paired-end on either an Illumina HiSeq 2000, NextSeq^TM^ 500, or NovaSeq 600 (~25 million reads per sample).

Transcript expression was quantified using Kallisto (v.46.2) [36] with GRCh38/hg38 as reference transcriptome, and subsequently aggregated to gene level using tximport (v1.16.1) [37]. Trimmed Means of M-values (TMM) normalisation, filtering, and log2 transformation was performed on gene level counts using edgeR (v.3.30.3) [38]. Batch effect correction was performed using the removeBatchEffect function in the R package Limma (v3.44.3) [39], and batch effect adjusted data was used for subsequent analyses.

Relative cell-type enrichment of CD8^+^ T-cells was estimated from the total RNA sequencing data using xCell in R(v.1.1.0) [40]. xCell is a gene expression signature-based approach to determine cell-type enrichment from bulk tissue RNA expression data, used here to explore the enrichment of a CD8^+^ T-cell signature. High expression of a specific signature results in a high enrichment score for the corresponding cell type.

### Spatial transcriptomics and data analysis

Spatial transcriptomics (ST) was performed on three representative prostate tissue samples from three patients included in the MPC cohort. An experienced pathologist confirmed the presence of malignant lesions within the tissue sections based on H&E staining. ST was performed on an adjacent tissue section (10 μm) using the Visium spatial gene expression methodology following the manufacturer’s instructions (10X genomics, Pleasanton, California, USA, #CG000239). A detailed description of the ST methodology is available elsewhere [41]. The 10X Visium spatial gene expression slide applies thousands of 55-μm barcoded spots within a 6.5 × 6.5 mm area to capture mRNA. The captured mRNA was sequenced on an Illumina NextSeq platform. Space Ranger (10X genomics) pipeline was used to align the reads to the human reference genome (hg38) and for obtaining the coordinates for each transcript. Data analysis was performed in R [42] using the Seurat package [43]. Spots with less than three detected genes were filtered out, and the data were normalised. The presence or absence of CD8A (CD8 alpha chain) and FOLH1 (PSMA) was determined using this dataset and visualised using the SpatialDimPlot function. Venn diagrams were generated using the ggvenn [44] R package.

### Statistical analysis

Recurrence-free survival analysis was conducted using the survminer package [45] (v.0.4.8) in RStudio (v. 1.0.153) using the Kaplan–Meier methods. Log-rank tests were used to determine significance with the clinical endpoint being biochemical recurrence (BCR), defined as PSA ≥ 0.2 ng/mL following RP. *P*-values below 0.05 were considered significant. PSMA expression and CD8^+^ T-cell infiltration in prostate tissue samples were compared with Wilcoxon rank-sum test [46]. When patients were dichotomised (high/high versus remaining), comparisons to clinical parameters (BCR, surgical margin status, pre-surgery PSA, CAPRA-S risk Score, T-stage and Gleason grade) were conducted using Fisher’s exact test [47]. To determine the correlation between PSMA expression and CD8^+^ T-cell infiltration, Pearson’s correlation coefficient [48] was calculated.

Experimental data were analysed using the Origin 2018 software and GraphPad Prism software v.8. The respective used statistical analysis is indicated in the corresponding figure and table legends. A minimum value of *P* < 0.05 was considered statistically significant. All experiments were conducted at least two times.

### External PC patient dataset

For external validation, the Prostate adenocarcinoma (PRAD) tumour RNA-seq dataset from The Cancer Genome Atlas (TCGA) [49] was downloaded from the TCGA data portal [50], as described previously [51]. PSMA (FOLH1) expression data and clinical data were available for 498 LPC and 52 AN prostate tissue specimens.

### Cell lines and culture conditions

LNCaP (ATCC, CRL—1740), Du-145 (ATCC, CRL—HTB—81), PC3 (ATCC, CRL—1435), C4-2 (ATCC, CRL—3314), HEK293E (ATCC, CRL—10852) and Jurkat cells (ATCC, TIB-152) were cultured in RPMI medium 1640 (Gibco, Waltham, Massachusetts, USA #61870-010) supplemented with 10% foetal bovine serum (FBS) (Gibco, #10500-064) and 1% penicillin–streptomycin (Gibco, #15140-122). Human dermal microvascular endothelial cells (HMEC-1) stably overexpressing human FcRn (HMEC-1-FcRn) [18] were cultivated in complete medium consisting of MCDB131 (Life Technologies, Waltham, Massachusetts, USA #10372-019), Recombinant human EGF (PeproTech, Cranbury, New Jersey, USA #AF-100-15), Hydrocortisone (Sigma-Aldrich, St. Louis, Missouri, USA #H0888), G418 solution (Sigma-Aldrich, #G418-RO), Puromycin (Life Technologies, #A11138-03), 10% FBS, and 2 mM l-glutamine (Lonza, Basel, Switzerland #BE17-605E). Cells were cultivated following ATCC protocols, and mycoplasma tested when new stock were thawed. Cell lines were routinely authenticated by short tandem profiling.

### Morphological characterisation by atomic force microscopy (AFM)

AlproTox (WT, NB, or HB) solution (1 µg/ml) was deposited on the positively charged mica surface, pretreated by (3-aminopropyl) triethoxysilane vapour (APTES; Sigma-Aldrich, #919-30-2), and, scanned in peak force tapping mode in the fluid using a MultiMode VIII (Bruker, Santa Barbara, California, USA) by SNL-10C (Bruker) cantilever with a spring constant of 0,24 N/m and a tip radius of 2 nm. All parameters were optimised during imaging to avoid protein deformation. Data were processed using SPIP (Image Metrology, Lyngby, Denmark).

### Neonatal Fc receptor engagement kinetics and cellular recycling

Bio-Layer Interferometry studies were conducted using the Octet Red96 system (FortéBio, Fremont, California, USA). Biotinylated human FcRn (hFcRn) (Immunitrack, Copenhagen, Denmark # ITF02) was immobilised on streptavidin-coated biosensors (Sartorius AG) in 0.01% PBST, pH 7.4. Wild-type (WT), null-binding (NB) or high-binding (HB) recombinant human albumin (rHSA) variants were used as controls for the albumin fusions. Kinetic measurements were performed in 25 mM NaOAc, 25 mM NaH_2_PO_4_, 150 mM NaCl, 0.01% PBST, pH 5.5 with sevenfold serial dilutions at maximum 3 µM concentration. A baseline was established in buffer before sensors were transferred to buffer containing analyte for 300 s followed by 600 s of disassociation in sample-free association buffer and 240 s of regeneration in PBST. Data analysis was performed using the Octet data analysis software ver. 10.0.1.6 (FortéBio) using a 1:1 binding model for curve fitting to estimate the kinetic parameters with all data referenced with FcRn-streptavidin sensors in wells containing only buffer.

Cellular recycling of the AlproTox or rHSA panel (0,1 µM) was performed in HMEC-1-FcRn overexpressing cells using a cellular recycling assay previously described [18].

### Cellular antigen recognition

LNCaP, C4-2, Du-145, PC3 or Jurkat cells were cultivated, and 1 × 10^5^ cells were seeded into a U-bottom Nunclon^TM^ plate and incubated on ice with dilution series of ice-cold assay buffer (PBS + 1% FBS) supplemented with either AlproTox, mouse anti-human CD3 FITC-conjugated antibody (Immunotools, Friesoythe, Germany #21850033), or PE-conjugated mouse monoclonal anti-PSMA antibody (Abcam, #ab77228). Following incubation, cells were pelleted and washed two times in 200 µL assay buffer before the addition of secondary antibody: rabbit polyclonal antibody to human albumin (Abcam, #ab2406). Plates were incubated and the wash routine was repeated before the tertiary antibody, goat anti-rabbit IgG FITC-conjugated antibody (Invitrogen, #A11034), was added with subsequent 30 min of incubation on ice. Cells were washed and resuspended in 200 µL 1:200 7-AAD live/dead cell stain (Invitrogen, Waltham, Massachusetts, USA #S10274) before samples were run on a Novocyte flow cytometer (ACEA Biosciences Inc, Santa Clara, California, USA). Control wells consisted of secondary and tertiary antibody. Data analysis was performed using FlowJo™ v10.0.7 Software (BD Life Sciences, Franklin Lakes, New Jersey, USA).

### T-cell activation and CD69 upregulation

Anti-human CD3 antibody clone OKT3 (Biolegend, Waltham, Massachusetts, USA #317302) was added to a 96 Nunc MicroWell plate (Thermo Fisher Scientific, Waltham, Massachusetts, USA #163320) at 10 µg/mL and left overnight at 4 °C. In all, 4 × 10^4^ LNCaP, C4-2, Du-145 or PC3 cells were seeded in triplicates together with a serial dilution of the AlproTox panel at a maximum 100 nM together with 2 × 10^5^ Jurkat cells. As a positive control, Jurkat cells seeded in the OKT3-coated wells was used. Following 24 h of incubation, cells were spun down and resuspended in 50 µL assay buffer (PBS + 1% FBS) before washing and addition of 100 µL anti-CD69 FITC (ImmunoTools, #21620693×2) at 1:100 dilution. Cells were incubated at 30 min, before 2× washing and resuspending in 200 µL 1:200 7-AAD before running the samples on a Novocyte flow cytometer (ACEA Biosciences Inc). Data analysis was performed using FlowJo™ v10.0.7 Software (BD Life Sciences).

### LDH cytotoxicity assay

In total, 4 × 10^5^ target cells (LNCaP, C4-2, Du-145 or PC3) were seeded onto 96-well TC-treated flat-bottomed plate and incubated at 37 °C, 5% CO_2_ for 24 h. Freshly isolated PBMCs from healthy donors were added at a 5:1 concentration to target cells along with a dilution series of AlproTox WT, NB or HB at maximum 100 nM in assay media (RPMI 1640 + Glutamax, 10% FBS, 1% penicillin–streptomycin) and co-incubated for 48 h. Separate experimental repeats used different PBMC donors. 1% final concentration Triton X-100 (Cell Biolabs Inc, San Diego, California, USA #124102) was added to high control wells for 15 min for cell lysis verified by light microscopy before spinning down cells at 600×*g* and transferring 100 µL supernatant to a 96-well Nunc MaxiSorp^TM^ flat-bottomed plate in duplicates for a total of two technical replicates for each experimental repeat. As low control, untreated wells containing target and effector cells only were used. The LDH cytotoxicity detection kit (TaKaRa, Kusatsu, Japan #MK401) was used according to the manufacturer’s instructions. Absorbance was measured at 620 nm with 492 nm as background by a CLARIOstar^TM^ (BMG LABTECH, Ortenberg, Germany). Cytotoxicity was calculated following the equation, cytotoxicity (%) = ((experiment value – low control)/(high control – low control)) × 100.

### Reporting summary

Further information on research design is available in the Nature Research Reporting Summary linked to this article.

## Results

### CD8^+^ T-cell infiltration and PSMA expression in prostate tumours

Total RNA sequencing data from seven normal (N) prostate tissue samples, 31 adjacent-normal (AN) prostate tissue sample as well as primary PC tissue samples from 126 patients with clinically localised PC (LPC) and 17 patients with metastatic PC (MPC) was used for quantification of PSMA expression. In addition, the xCell algorithm was applied to the RNA-seq data for cell-type enrichment analysis to estimate the levels of infiltrating CD8^+^ T-cells. To visualise the spatial distribution of CD8A + (gene encoding CD8) and FOLH1 + (gene encoding PSMA) spots, we performed ST on three representative prostate tissue samples from the MPC patient cohort. For external validation, we used RNA-seq data from the TCGA-PRAD cohort, including 498 LPC samples and 52 AN samples. For patient characteristics, refer to Supplementary Table 1.

**Fig. 1:**
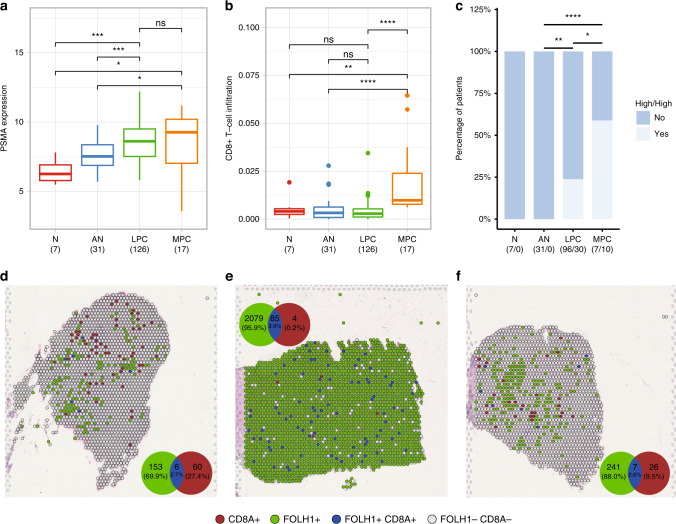
PSMA expression and CD8^+^ T-cell infiltration in non-malignant and prostate cancer tissue samples. **a** PSMA expression, **b** CD8^+^ T-cell infiltration and **c** proportion of patients having high PSMA expression and high CD8^+^ T-cell infiltration (High/High) defined as >median PSMA expression and >median CD8 infiltration. N = normal, AN = adjacent normal, LPC = tumour tissue from patients with localised PC, MPC = primary tumour tissue from patients with metastatic prostate cancer. (**a**–**b** (*n*) = number of patients, **c** (*n/n*) = number of patients locating to Remaining/number of patients locating to High/High). PSMA expression shown using log2 fold change while CD8^+^ T-cell infiltration shown as cell enrichment score. **d**–**f** Spatial transcriptomics on three representative samples from the MPC cohort, with (**d**) corresponding to remaining subset and **e**, **f** corresponding to High/High subset, showing spatial distribution of CD8A + (red), FOLH1 + (green), and CD8A + FOLH + (blue) spots. Venn diagram depicts overlap between CD8A + and FOLH1 + spots. Statistical analysis was conducted in Rstudio (Wilcoxon test (**a**, **b**), Fisher’s exact test (**c**): *<0.05, **<0.01, ***<0.001, ****<0.0001, ns non-significant).

PSMA was overexpressed in both MPC and LPC samples, as compared to AN prostate tissue samples (*P* = 0.018 and *P* < 0.001 respectively, Wilcoxon test, Fig. 1a), and as compared to normal (N) prostate tissue samples (*P* = 0.011 and *P* < 0.001, respectively, Wilcoxon test, Fig. 1a). There was no significant difference in PSMA expression between LPC and MPC samples in this patient cohort (*P* = 0.39, Wilcoxon test, Fig. 1a). PSMA was also overexpressed in LPC as compared to AN samples in the TCGA dataset (*P* < 0.001; Wilcoxon test; Supplementary Fig. s1a).

CD8^+^ T-cell enrichment score was increased in MPC samples as compared with all other sample types analysed (*P* < 0.05, Wilcoxon test, Fig. 1b). There was no significant difference in CD8^+^ T-cell enrichment score between N, AN and LPC samples (*P* > 0.05, Wilcoxon test, Fig. 1b). Similarly, there was no significant difference in CD8^+^ T-cell enrichment between AN and LPC samples in the external TCGA cohort (*P* > 0.05; Wilcoxon test; Supplementary Fig. s1b). There was no significant correlation between overall PSMA expression and CD8^+^ T-cell infiltration in MPC or LPC samples (*P* = 0.17 and *P* = 0.3, respectively; Pearson, Supplementary Fig. s2), and a similar result was found in the TCGA dataset (Supplementary Fig. s3).

A focus was to identify patients with a high PSMA expression and a high CD8^+^ T-cell infiltration profile as potential candidates for bispecific T-cell engager treatment. Hence a score designated High/High defined as > median PSMA expression and > median CD8^+^ T-cell infiltration was determined (cut-offs based on median in tumour samples from LPC patients). 59% of patients with MPC (*n* = 10/17) scored as High/High, as well as 24% of patients with LPC (*n* = 30/126) (Fig. 1c). Similarly, in the TCGA cohort 25% (*n* = 126/498) of patients with LPC scored as High/High (Supplementary Fig. s1c).

ST validated the presence of CD8^+^ cells in the prostate tissue from all three MPC patients (Fig. 1d–f), corroborating the enrichment of CD8^+^ T-cells (xCell) as identified from bulk tumour RNA-seq in this study. FOLH1 (PSMA) expression was heterogeneous in the analysed tissue but seemed predominant in the High/High samples with total proportion of FOLH1+ spots being 91.8% (e) and 17.1 % (f) compared to the 9.1% in tissue samples belonging to remaining group (d). Whilst the total overlap between FOLH1+ and CD8A+ spots appears low (2.6–3.9%), the proportion of double positive spots, with respect to CD8A+ spots, was considerable (9.1–95.5%). Furthermore, the two largest overlaps were found in the High/High subgroup, 95.5% (e) and 21.2% (f), while the lowest overlap was seen in the remaining group, 9.1% (d).

### Association of CD8^+^ T-cell infiltration and PSMA expression with high-risk prostate cancer

Next, we assessed the prognostic value of having both high CD8^+^ T-cell infiltration and high PSMA expression in LPC patients (High/High phenotype, *n* = 30). The remaining patients (*n* = 96), with below median expression of PSMA and/or below median CD8^+^ T-cell infiltration, were assigned to a separate group.

**Fig. 2:**
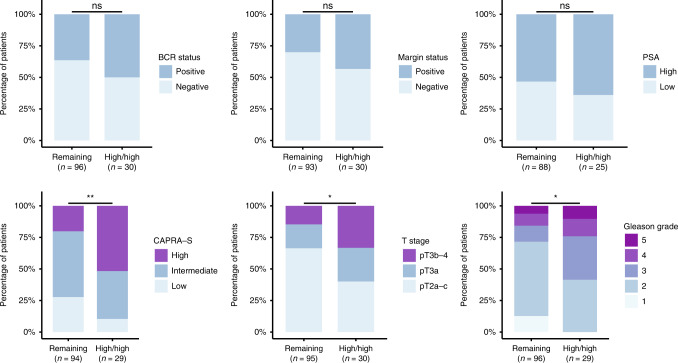
Prostate cancer characteristics in relation to PSMA expression and CD8^+^ T-cell infiltration in LPC tissue samples from 126 patient who have undergone radical prostatectomy. Patients were stratified (remaining versus High/High) based on the median PSMA expression and median CD8^+^ T-cell enrichment score, with patients scoring above median in both categories being placed into High/High. *n* = number of patients. Statistical analysis was conducted in Rstudio (Fisher’s exact test: *<0.05, **<0.01, ***<0.001, ****<0.0001, ns non-significant).

Biochemical recurrence status (BCR, defined as PSA > 0.2 ng/mL), surgical margin status, and pre-surgery serum PSA levels (PSA < 10 ng/mL = low, PSA ≥ 10 ng/mL = high) were not statistically different between the two groups (Fisher’s exact test, *P* > 0.05, Fig. 2). Kaplan–Meier analysis of BCR-free survival also did not show significant differences between the two subgroups (Supplementary Fig. s4). Similar results were observed in the TCGA cohort (Supplementary Figs. s5 and s6).

However, for all these factors, there was a moderate trend towards more high-risk disease characteristics in the High/High subgroup. In support of this, there was a significant association between the High/High subgroup and more advanced T stage (*P* = 0.025, Fisher’s exact test) as well as a significant association with higher Gleason Grade *(P* = 0.0143, Fisher’s exact test, Fig. 2). To improve statistical power, we used the established clinical risk-stratification tool, USCF Cancer of the Prostate Risk Assessment Post-Surgical (CAPRA-S) score [52], to categorise the patients into a high-, intermediate-, and low-risk PC subgroup. Here, there was a significant association between a higher CAPRA-S score and the High/High phenotype, defined by high PSMA expression and high CD8^+^ T-cell infiltration (*P* = 0.004, Fisher’s exact test). These findings were also supported in the TCGA cohort, where the High/High phenotype was significantly associated with a higher CAPRA-S risk score (*P* < 0.001, Fisher’s exact test, Supplementary Fig. s6) and higher Gleason Grade (*P* < 0.001, Fisher’s exact test; Supplementary Fig. s6) as well as borderline associated with advanced T stage (*P* = 0.099, Fisher’s exact test, Supplementary Fig. s6). To determine if CD8^+^ T-cell infiltration correlated to clinical parameters independently of PSMA expression, LPC patients were stratified into a High vs. Remaining subgroup, based solely on median CD8^+^ T-cell infiltration. No clinical parameters were significant for the LPC cohort, although the same trend towards more high-risk disease characteristics, as seen for the High/High versus Remaining subset (Fig. 2), was apparent (Supplementary Fig. s7). For the larger TCGA cohort, higher CAPRA-S risk score (*P* = 0.044, Fisher’s exact test), higher Gleason Grade (*P* < 0.001, Fisher’s exact test) and positive margin status (*P* = 0.038, Fisher’s exact test) were all significantly associated with high CD8^+^ T-cell infiltration (Supplementary Fig. s8).

In summary, results from two independent PC patient cohorts indicate that a subset of patients with localised and metastatic disease express high levels of PSMA and have high CD8^+^ T-cell infiltration levels (High/High phenotype). The High/High phenotype seemed to be common in advanced MPC. Furthermore, the patients with localised disease in this subset had clinical characteristics known to be associated with higher-risk cancer, providing rational for treatment with bispecific T-cell engagers following radical prostatectomy.

### Expression of AlproTox

AlproTox vector constructs containing an anti-CD3 × anti-PSMA bispecific antibody were genetically fused to the N-terminus of wild-type (WT) HSA (AlproTox WT), or mutated HSA variants for null-binding (NB) (AlproTox NB) or high-binding (HB) (AlproTox HB) to FcRn. These were transiently transfected into HEK293E cells and cultivated in serum-free media. The AlproTox fusions were purified using affinity chromatography (Supplementary Fig. s9) and collected eluate fractions were analysed with ELISA (Supplementary Fig. s10). Final samples analysed with Coomassie staining (Supplementary Fig. s10), and western blotting confirmed high purity of samples (Supplementary Fig. s11). Protein yields of 0.59 mg/L, 0.76 mg/L and 0.54 mg/L were obtained for AlproTox WT, NB and HB, respectively.

### Morphology, FcRn affinity and cellular recycling

**Fig. 3:**
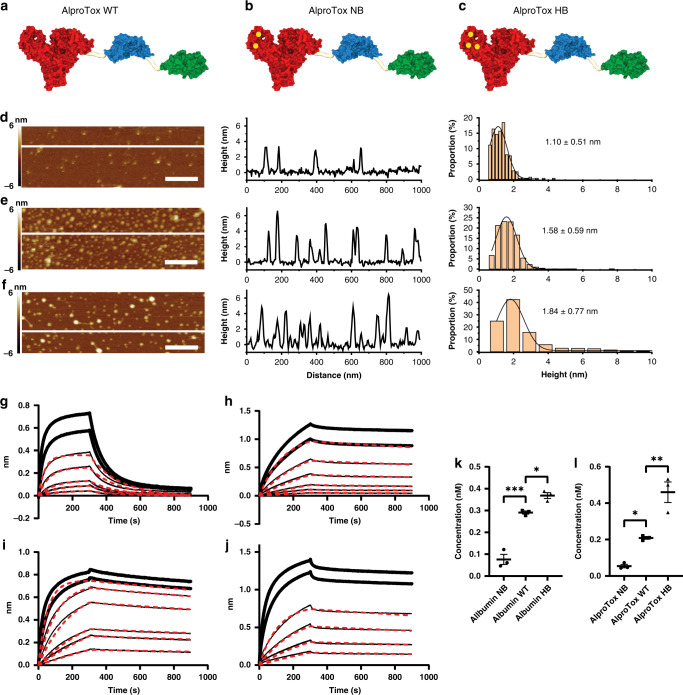
AlproTox morphology, binding kinetics and cellular recycling. **a**–**c** Schematic representation of the AlproTox panel containing an anti-PSMA human domain antibody (Green) linked to an anti-CD3 scFv (Blue) and HSA (Red) with amino acid point mutations (yellow dots) providing different FcRn affinity (NB: K500A, H510Q, HB: E505Q, T527M, K573P). **d**–**f** Representative AFM images of particle height and histograms showing distribution and average height of AlproTox WT, NB and HB, respectively. Scale bar is 200 nm and intensity scale −6–6 nm. **g**–**j** Sensorgrams showing binding of serial dilutions of protein to immobilised human FcRn at pH 5.5. Red curves display fit. **g** Binding curves of albumin WT. **h** Binding curves of AlproTox WT. **i** Binding curves of albumin HB. **j** Binding curves of AlproTox HB. **k** rHSA detection in the supernatant following a cellular recycling assay. **l** AlproTox detection in the supernatant following a cellular recycling assay. Statistical analysis was made in Graphpad prism v.8 using ANOVA one-way with Tukey Post hoc correction. Mean ± SEM shown, *N* = 3 independent experiments.

The panel of fusions (Fig. 3a–c) displayed uniform discrete particle characteristics of similar nanoscale size ranging from: 1.10 ± 0.51 nm, 1.58 ± 0.59 nm, and 1.84 ± 0.77 nm for AlproTox WT, AlproTox NB and AlproTox HB, respectively (Fig. 3d–f). The AlproTox HB and WT fusions displayed a clear pH-dependent affinity towards human FcRn (hFcRn) (K_D_ 5.3 × 10^−9 ^M and K_D_ 1.1 × 10^−8 ^M, respectively) with a profile comparable to that of recombinant HB and WT HSA non-fused albumin controls (Fig. 3g–j). Both AlproTox HB and AlproTox WT, as well as the respective recombinant HSA (rHSA) variant counterparts, could be fitted to a 1:1 binding model with results displayed in Table 1. AlproTox NB and HSA NB, as expected, could not be fit to a 1:1 binding model, and as such are not shown.

**Table 1:** Measured binding affinities of rHSA and AlproTox variants to hFcRn (K_D_, K_on_ and K_off_) at pH 5.5.

	K_D_ (M)	K_on_ (1/ms)	K_off_ (1/s)	R^2^ 1:1 fit	X^2^ fit
Albumin NB	–	–	–	–	–
Albumin WT	2.1E-07	2.8E + 04	5.8E-03	0.979	0.788
Albumin HB	7.7E-09	2.5E + 04	1.83E-04	0.997	0.865
AlproTox NB	**–**	–	–	–	–
AlproTox WT	1.1E-08	1.8E + 04	1.96E-04	0.998	0.853
AlproTox HB	5.3E-09	5.0E + 04	2.6E-04	0.9982	0.459

Data were obtained by Bio-Layer Interferometry using an Octet system. Sensors were coated with hFcRn and lowered into wells containing the analyte. For affinity analyses, the sensorgrams were fitted to a 1:1 model.

The level of AlproTox recycled in endothelial HMEC-1-FcRn cells correlated with measured hFcRn affinity, following the trend AlproTox HB (0.460 nM) > AlproTox WT (0.2090 nM) > AlproTox NB (0.054 nM) (Fig. 3k–l).

### Cellular antigen recognition

**Fig. 4:**
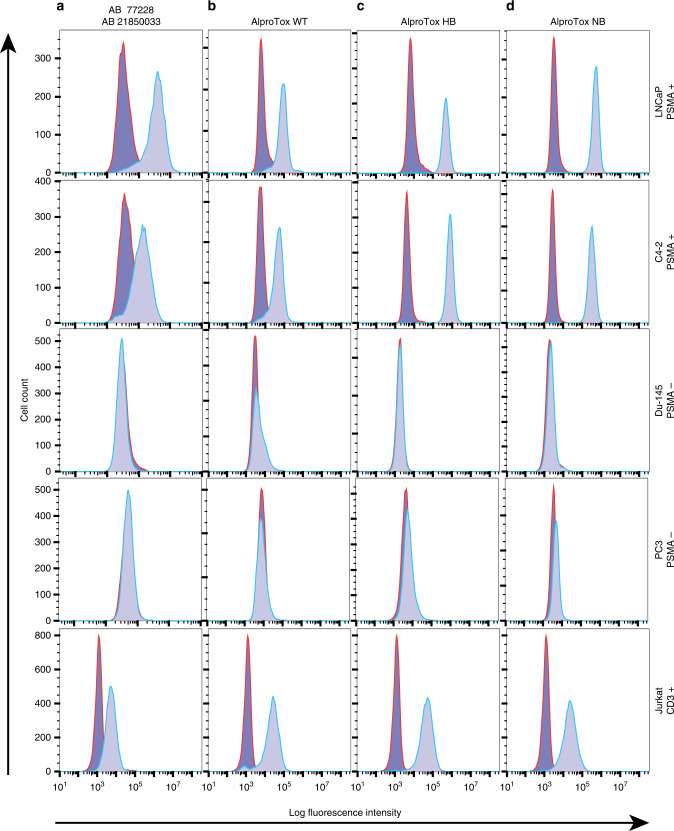
Flow cytometric analysis. It shows the panel of AlproTox WT, HB and NB fusions antigen-specific binding at 1 µg/mL in PSMA-positive (LNCaP, C4-2) and CD3-positive cells (Jurkat) detected by an anti-human serum antibody (**b**–**d**). No shifts in fluorescence were detected in non-expressing CD3 or PSMA (PC3, Du-145) cells. Secondary antibody without primary antibody was used to determine background intensity. PSMA and CD3 expression was verified (**a**) with an anti-PSMA PE-conjugated antibody (ab77228) and an anti-CD3 FITC-conjugated antibody (21850033), respectively.

The AlproTox panel bound specifically to PSMA-expressing PC cell lines (LNCaP, C4-2) and CD3 expressing T-lymphocyte (Jurkat) cells at concentrations of 1 µg/mL demonstrated by a shift in fluorescent intensity using commercial antibodies (Fig. 4). No binding was detected for the PSMA negative PC cell lines (PC3, Du-145). The AlproTox fusions displayed similar shifts in fluorescence, independent of hFcRn affinity, and shifts in fluorescence were detected only for PSMA and CD3 expressing cell lines, demonstrating binding specificity.

### T-cell activation and T-cell-mediated cytotoxicity

**Fig. 5:**
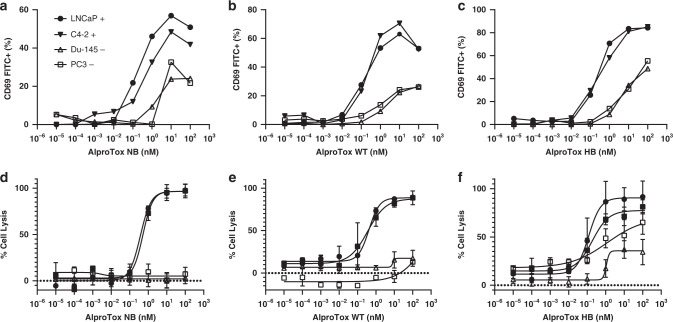
T-cell activation and T-cell-mediated cell killing by AlproTox. An increasing dose of tested AlproTox fusion was co-incubated with Jurkat cells and PC cell lines (PSMA-positive closed symbols, PSMA negative open symbols) followed by flow cytometry analysis quantifying the expression of CD69 (**a**–**c**) Cell killing was quantified using an LDH-endpoint assay determining the percentage of lysed PC cell lines when co-incubated with increasing levels of AlproTox and isolated PBMCs (**d**–**f**). Statistical analysis was made in Graphpad prism v.8 with Mean ± SEM shown, *N* = 3 independent experiments, (LNCaP *N* = 2 independent experiments).

CD69 expression as a measure of T-cell activation strongly correlated with the concentration of AlproTox and PSMA expression on the target cells. The target cells displayed concentration-dependent cell lysis when incubated with AlproTox WT, NB or HB in the presence of PBMCs, whilst no cell lysis was detected for PSMA negative cell lines incubated with AlproTox WT or NB (Fig. 5d–f). The EC_50_ ranged from 0,44 nM (NB), 0.39 nM (WT) to 0,16 nM (HB), with AlproTox HB displaying some degree of cell lysis of the PSMA negative Du-145 and PC3 cell lines (Fig. 5f).

## Discussion

Bispecific T-cell engagers that drive redirection of the effector T-cell response, provides a potential strategy to overcome the multiple resistance mechanisms seen in advanced PC [9, 30]. However, the investigation into selecting PC patients most likely respondent to T-cell engagers is lacking and is one of the main hurdles to bispecific T-cell engager adoption.

In this study, we aimed to identify patients who could be candidates for T-cell engager immunotherapy and introduce a novel anti-CD3 x PSMA albumin-fused bispecific T-cell engager with tunable FcRn-driven cellular recycling for programming the circulatory half-life and consequent duration of effect. RNA sequencing of tumour samples from PC patients showed increased PSMA expression in the prostate of PC-diagnosed patients and increased CD8^+^ T-cell infiltration in patients with metastatic disease (Fig. 1a–c). Whilst the clear prognostic value of PSMA is established [23], it is not the case for T-cell infiltration. Some studies associate CD8^+^ T-cell infiltration to longer biochemical recurrence-free survival [53], others finding no significant effect [54], and others like Ness et al. finding clear association with shorter biochemical recurrence-free survival following radical prostatectomy [55]. *Hitherto*, no studies have investigated the co-localisation of PSMA and CD8^+^ T-cell infiltration in PC tumours and the combined impact on disease aggressiveness and biochemical recurrence in localised disease patients. From the cohort of patients investigated, we found a subgroup of MPC patients (59%, *n* = 10/17), expressing high PSMA and high CD8^+^ T-cell infiltration in the primary tumour. ST analysis confirmed CD8^+^ cell infiltration in MPC prostate with 9.1–95.5% of CD8A+ positive spots overlapping with FOLH1+ spots, indicating the proximity of effector T-cells and PSMA-expressing cancer cells in the tumour tissue (Fig. 1d–f). Unfortunately, it was not possible to supplement the ST images with immunohistochemistry for single-cell image resolution to verify spatial location. However, The ST images alone suggest clinical utility for bispecific T-cell engagers that are reliant on the formation of an immunological synapse between cancer and T cell, and, thus, require direct cell-cell interaction. Furthermore, the motility of infiltrating CD8^+^ T-cells within tumour tissue [56] may allow for sequential cell-cell interaction in a high PSMA-expressing environment, predominant in the High/High subset. A total of 24% (*n* = 30/126) of LPC patients exhibited a similar High/High profile and were stratified according to these parameters with the High/High subgroup showing significantly higher CAPRA-S Score, advanced T stage and higher Gleason grade, indicative of a high-risk form of prostate cancer (Fig. 2). The findings were validated in an independent PC tumour RNA-seq dataset (TCGA-PRAD) [49], where a similar High/High subgroup was identified (25%, *n* = 126/498, Supplementary Fig. s1), that also exhibited significantly higher CAPRA-S Score, (borderline) T stage and Gleason grade (Supplementary Fig. s6). A similar association with clinical parameters was found for the TCGA cohort when stratified only on CD8^+^ T-cell infiltration albeit with higher *P*-values for all parameters except margin status, which was now significant (Supplementary Fig. s8). For the LPC patients, however, no significant associations with the clinical parameters were found (Supplementary Fig. s7), indicating that the combination of PSMA expression and CD8^+^ T-cell infiltration can more accurately identify the high-risk patient subtype. The inconclusive influence on tumour characteristics and inconsequential effect on BCR of tumour infiltrating CD8^+^ T-cells is in line with the inconsistent nature of prior study results on the subject [57]. T-cell infiltration has been shown correlated to mutational burden in multiple cancers, with high mutational burden as well as mutations in mismatch repair genes correlating to high T-cell infiltration [58, 59]. While genomic analysis is beyond the scope of this paper, further characterisation of the identified high-risk prostate cancer subset in regard to specific mutations and mutational load are warranted and should be included in future studies. Current European clinical guidelines [60] recommend multimodal treatment of localised high-risk prostate cancer and ADT, commonly used to treat locally advanced and metastatic cancer, has been shown to cause an influx of CD8^+^ T-cells in the primary tumour [61]. This could, in part explain the higher level of CD8^+^ T-cell infiltration seen in the prostate tissue of the MPC patients when compared to the LPC patients (Fig. 1) that were all treatment naïve prior to RP. The impact of immunotherapy in combination with ADT on treatment efficacy is currently being evaluated in a Phase II/III clinical trial of metastatic patients [62], and whilst metastatic patients should indeed be considered the highest treatment priority for novel drugs, the identified subset of LPC patients (High/High) also stand to benefit from additional treatment and may be responsive to treatment with an anti-CD3 x PSMA bispecific T-cell engager.

A limitation of the study was the inability to examine anti-tumour efficacy based on pretreatment CD8^+^ T-cell infiltration. While it is plausible that the localised presence of readily available CD8^+^ T-cells may potentiate treatment with bispecific T-cell engagers in solid tumours, this hypothesis has only been evaluated in a few studies of non-haematological malignancies. Ströhlein et al. found that relative lymphocyte count was a direct positive prognostic parameter in peritoneal carcinomatosis patients treated with catumaxomab, a CD3 targeting bispecific antibody, in a Phase II clinical trial [63]. In support of this, Belmontes et al. found that the most important factor for bispecific T-cell engager efficacy in multiple solid-tumour in vivo models was pretreatment T-cell density in the tumour, with CD8^+^ T-cells being the most important mediator of bispecific T-cell engager activity [14]. Finally, although based on different classes of immunotherapeutics, parallels can be drawn to immune checkpoint inhibitors that also rely on CD8^+^ T-cell cytotoxicity to mediate effect on which there is a more extensive body of literature. In a multiomics analysis of 21 types of cancer, including prostate adenocarcinoma, a study found that the best indicator, out of 36 variables, to anti-PD-1/PD-L1 therapy was CD8^+^ T-cell abundance in the tumour [64]. While the success of checkpoint inhibitors has been modest in the treatment of prostate cancer, Graff et al. in a small Phase II trial (*n* = 10) described two mCRPC patients that showed robust response to anti-PD-1 treatment with baseline tumours having CD8^+^ T-cell infiltration [65]. The higher rate of immunotherapeutic response rates seen in tumours with CD8^+^ T-cell infiltration, coupled with the heightened risk profile identified in our study, therefore, lends plausibility to the identified patient subset in our work being potential treatment candidates. However, further studies on a possible role of tumour infiltrating CD8^+^ T-cells in bispecific T-cell engager treatment would be interesting. Another limitation was the inability to explore the PSMA expression and CD8^+^ T-cell infiltration in metastasis biopsies, despite these sites being relevant targets for successful disease treatment. Both Queisser et al. and Wright et al. demonstrated increased PSMA expression in metastatic lesions compared to localised tumour [66, 67]. Furthermore, Sartor et al. demonstrated in their Phase III clinical trial of ^177^Lu-PSMA-617 that ~86.6% (*n* = 869/1003) of mCRPC patients imaged were PSMA-positive, indicative of the high prevalence of PSMA-positive lesions at later stages of PC [68]. Still, correlation with CD8^+^ T-cell infiltration was unknown [66, 67]. The results in our work, however, have identified, for the first time, a high-risk PC cohort, encompassing patients with localised and metastatic prostate cancer that could benefit from treatment with an anti-CD3 ×  PSMA bispecific engager.

While better stratification may improve therapeutic effect, inherent defects in bispecific T-cell engager design, namely short blood circulatory half-life, and adverse effects in the form of CRS are hindering clinical development. Despite Fc silencing, residual binding to the Fcγ receptor has been shown [69] and could potentially lead to overzealous immune stimulation. Furthermore, the large number of mutations required to silence the Fc domain may increase the risk of anti-drug antibody generation and protein instability [70]. Albumin is an attractive half-life extension alternative that has reached the clinic [19, 20] driven by FcRn-mediated cellular recycling [18] without induction of Fcγ innate immunity.

We introduce a novel anti-CD3 x PSMA albumin-fused bispecific T-cell engager with tunable FcRn affinity that may offer programmable pharmacokinetics (PK) and control of therapeutic effects. The panel of AlproTox fusions (null-binding (NB), wild-type (WT) or high-binding (HB)) were expressed in HEK293E cells with yields ranging from 0.54 mg/L to 0.76 mg/L. FcRn affinity was dependent on the incorporated albumin sequences shown previously to modulate FcRn binding [71], which followed the trend AlproTox HB (K_D_ 5.3 × 10^−9 ^M) > AlproTox WT (K_D_ 1.1 × 10^−8 ^M) > AlproTox NB. This correlated with levels recycled in the endothelial cellular recycling assay (Fig. 3k–l). Vasculature endothelial cellular recycling and concomitant extended circulatory half-life of albumin is mediated by an cellular endosomal sorting process diverting albumin from lysosomal breakdown facilitated by higher FcRn engagement at low endosomal pH and release at physiological pH at the cell surface [18]. Results from our group have shown that FcRn affinity and FcRn-driven cellular recycling correlates with the circulatory half-life of other bispecific (EGFR × CD3) albumin fusions with the inclusion of identical albumin sequences to the AlproTox fusions in this study [22]. It is, therefore, expected that the HB > WT > NB circulatory half-life trend will be observed in vivo with the AlproTox design. Specific binding against PSMA and CD3 was verified using flow cytometry (Fig. 4), with the panel exhibiting a similar binding profile. All albumin fusions were able to activate T cells when co-incubated with PSMA-expressing cell lines (Fig. 5a–c) and mediate T-cell cytotoxicity against PSMA-expressing prostate cancer cell lines (Fig. 5d–f). EC_50_ was within one order of magnitude across the panel, being highest for AlproTox NB (0.44 nM) and lowest for AlproTox HB (0.16 nM). The T-cell-mediated cell-killing efficacy is comparable to published AMG 160 results where EC_50_ values ranged from 6 to 42 pmol/mL, dependent on cell line [31]. While NB and WT were highly specific, cell killing was detected for AlproTox HB in PSMA negative cell lines, that may indicate binding towards endogenous FcRn expressed at low levels in the Du-145 and PC3 prostate cancer cell lines [72]. AFM analysis revealed discrete AlproTox constructs at the nanoscale that supports the possibility of these systems to penetrate solid tumours (Fig. 3d–f). Furthermore, exploitation of PSMA-targeting may mediate delivery to both primary sites and metastatic lesions.

Future work should investigate the PK in a physiologically relevant double transgenic human FcRn^+/+^/HSA^+/+^ mouse model introduced by our group [73] that abolishes competition from endogenous mouse albumin associated with wild-type strains. Furthermore, we have developed a *RAG1* knockout FcRn^+/+^/HSA^+/+^ strain that we have used to investigate T-cell engager efficacy [22] that should be applied to AlproTox anti-PC tumour investigations. The availability of a panel of HSA constructs with different PK may offer a tool to tune the dosage requirements and therapeutic effects. The well-documented entry of HSA at tumour sites by both passive [74] and active [75] processes also offers the advantage of improved accumulation at tumour sites.

In summary, we have investigated the PSMA expression and CD8^+^ T-cell infiltration in primary tumour samples of PC patients from two independent cohorts and identified a patient subset with high-risk local disease or metastatic cancer that may be susceptible to T-cell engager therapy. Precise stratification of patients may improve clinical response rates and, in combination with novel bispecific designs, lead to the successful adoption of bispecific T-cell engagers in PC.

## Supplementary information

Supplementary figures

Reporting Summary

Supplementary methods

## Data Availability

All data are available from the corresponding author upon reasonable request.

## Code availability

Code can be made available by making a request to the corresponding author.
