# Supplementary material for: Identification of a high-risk immunogenic prostate cancer patient subset as candidates for T-cell engager immunotherapy and the introduction of a novel albumin-fused anti-CD3 × anti-PSMA bispecific design
Source: Br J Cancer. 2022 Oct 15;127(12):2186–97. doi: 10.1038/s41416-022-01994-1 (PMC9727128; doi:10.1038/s41416-022-01994-1)
Supplement: Supplementary file 1 — Supplementary figures [file 41416_2022_1994_MOESM1_ESM.pdf]

	LPC<br>n = 126	TCGA<br>n = 498	Metastatic<br>n = 17
**Age at RP* (years)**			
Median (range)	65.1 (45-78)	61 (41-78)	66.5 (49.3-82)
**Pre-RP PSA (ng/mL)**			
Median (range)	10.6 (2-193)	7.5 (0.7-107)	-
Unknown, n (%)	13 (11.5 %)	37 (7.4 %)	-
**Gleason Grade Group****			
1	12 (9.52 %)	45 (9.0 %)	1 (5.8 %)
2	68 (54.0 %)	144 (28.9 %)	1 (5.8 %)
3	22 (17.5 %)	94 (18.9 %)	1 (5.8 %)
4-5	23 (18.3 %)	193 (38.8 %)	12 (70.6 %)
Unknown, n (%)	1 (0.79 %)	22 (4.4 %)	2 (11.7 %)
**RP T stage**			-
PT2a-c	75 (59.5 %)	184 (37.0 %)	-
T3a	26 (20.6 %)	152 (30.5 %)	-
T3b-4	24 (19.0 %)	133 (26.7 %)	-
Unknown, n (%)	1 (0.79 %)	29 (5.8 %)	-
**Margin status**			
Negative	82 (65.1 %)	304 (61.0 %)	-
Positive	41 (32.5 %)	137 (27.5 %)	-
Unknown, n (%)	3 (2.38 %)	57 (11.5 %)	-
**CAPRA-S Score**			
Low risk (0-2)	29 (27.0 %)	118 (23.7 %)	-
Intermediate risk (3-5)	60 (47.6 %)	166 (33.3 %)	-
High risk (≥ 6)	34 (23.0 %)	137 (27.5 %)	-
Unknown, n (%)	3 (2.38 %)	77 (15.5 %)	-
**Biochemical Recurrence**			
No	76 (60.3 %)	351 (70.5 %)	-
Yes	50 (39.7 %)	58 (11.6 %)	-
Unknown, n (%)	0 (0 %)	89 (17.9 %)	-
**Follow-up length (Months)**			
Median (range)	71 (2.9-204)	20.5 (3.1-151)	81.1 (8.4-183.6)
**Metastatic site**			
Local	-	-	1 (5.9 %)
Visceral	-	-	5 (29.4 %)
Local and Visceral	-	-	4 (24.5 %)
Unknown, n (%)			7 (41.18 %)
**Progression to CRPC prior to biopsy**			
No	126 (100 %)	498 (100 %)	11 (64.7 %)
Yes	0 (0 %)	0 (0 %)	5 (29.4 %)
Unknown, n (%)	0 (0 %)	0 (0 %)	1 (5.9 %)
**Therapy prior to biopsy**			
ADT	0 (0 %)	0 (0 %)	10 (58.8 %)
ADT and Antiresorptives	0 (0 %)	0 (0 %)	5 (29.4 %)
ADT and Chemotherapy	0 (0 %)	0 (0 %)	1 (5.9 %)
Curative radiation	0 (0 %)	0 (0 %)	1 (5.9 %)

Supplementary Table 1. Patient characteristics. CRPC = Castration resistant prostate cancer, ADT = Androgen deprivation therapy. *Age at TUR-P for metastatic patients, **Gleason grade group evaluated following RP for local cancer, and with TRUS biopsy for the metastatic cancer.

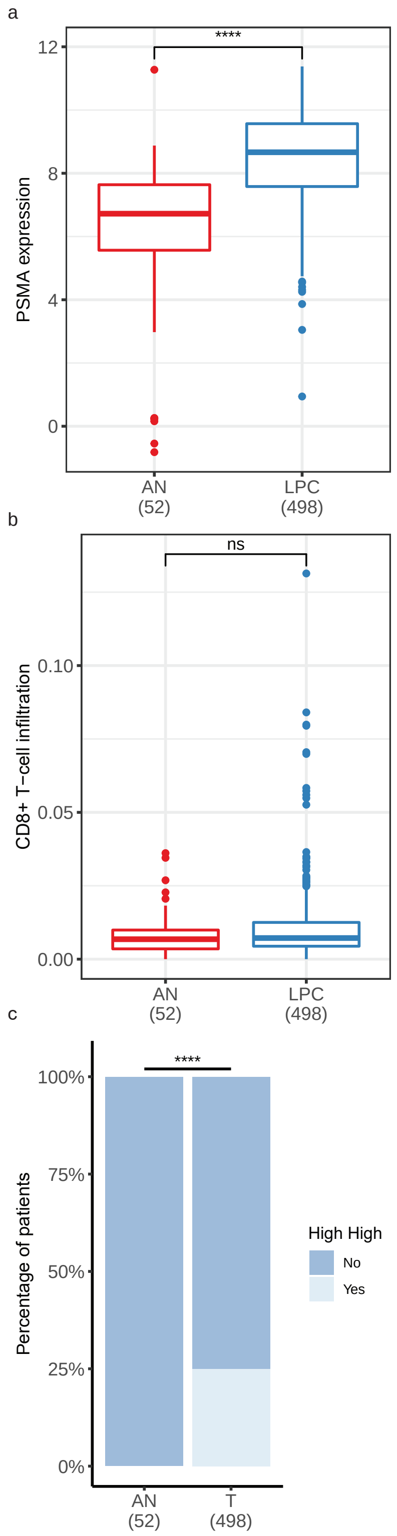

Figure s1. PSMA expression and $CD8^+$ T-cell infiltration in adjacent normal and prostate cancer tissue samples from PRAD-TCGA. **a** PSMA expression, **b** $CD8^+$ T-cell infiltration and **c** Proportion of patients having high PSMA expression and high $CD8^+$ T-cell infiltration (High/High) defined as > median PSMA expression and > median CD8+ infiltration. AN = Adjacent normal, LPC = Tumour tissue from patients with localised PC. PSMA expression shown using log2 fold change while $CD8^+$ T-cell infiltration shown as cell enrichment score. Statistical analysis was conducted in Rstudio (Wilcoxon test, panel a-b, Fisher's exact test panel c: *<0.05, **<0.01, ***<0.001, ****<0.0001, ns = non-significant)

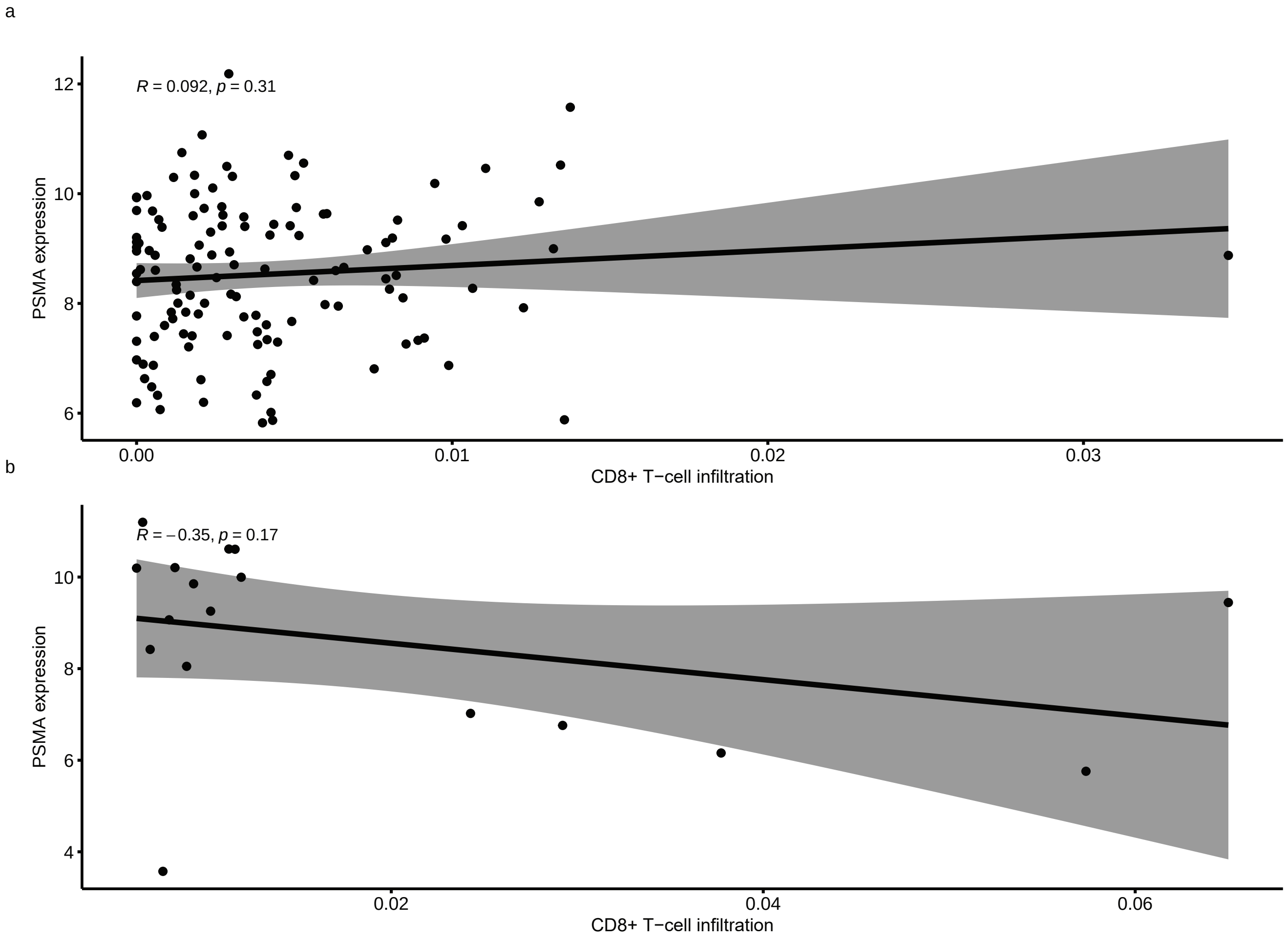

Figure s2. PSMA expression (Log2 fold change) and CD8[+] T-cell infiltration (enrichment score) in RP biopsies of **a** LPC and **b** MPC. Statistical analysis was conducted in Rstudio (Pearson correlation coefficient)

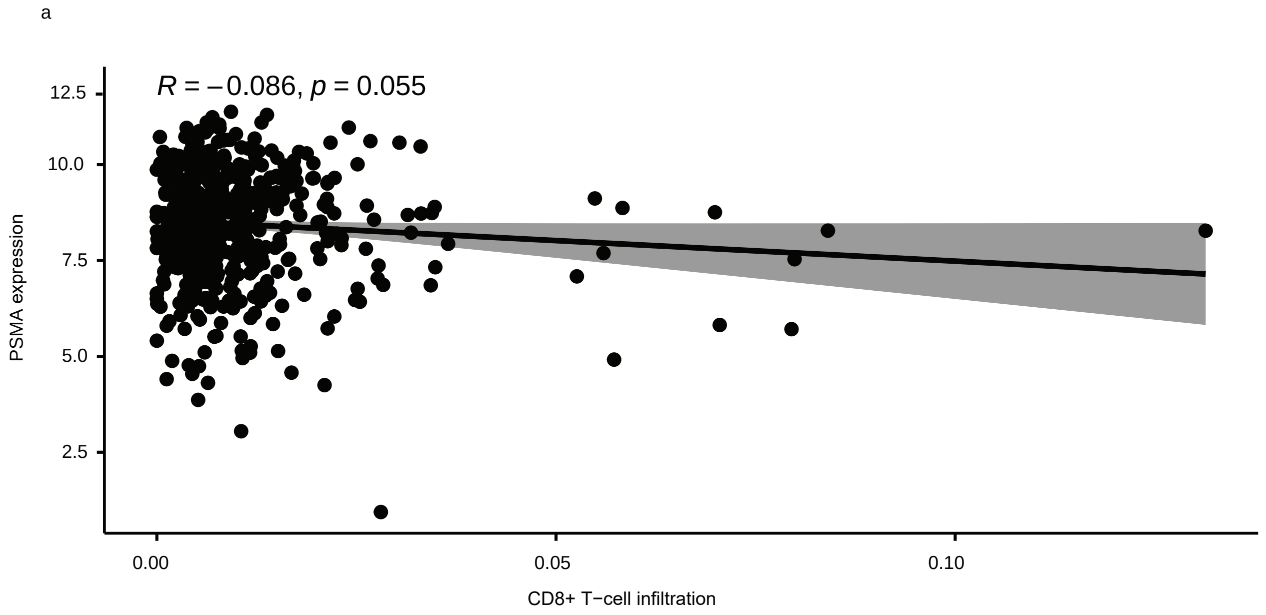

Figure s3. PSMA expression (Log2 fold change) and CD8[+] T-cell infiltration (enrichment score) in RP biopsies from the PRAD-TCGA dataset. Statistical analysis was conducted in Rstudio (Pearson correlation coefficient)

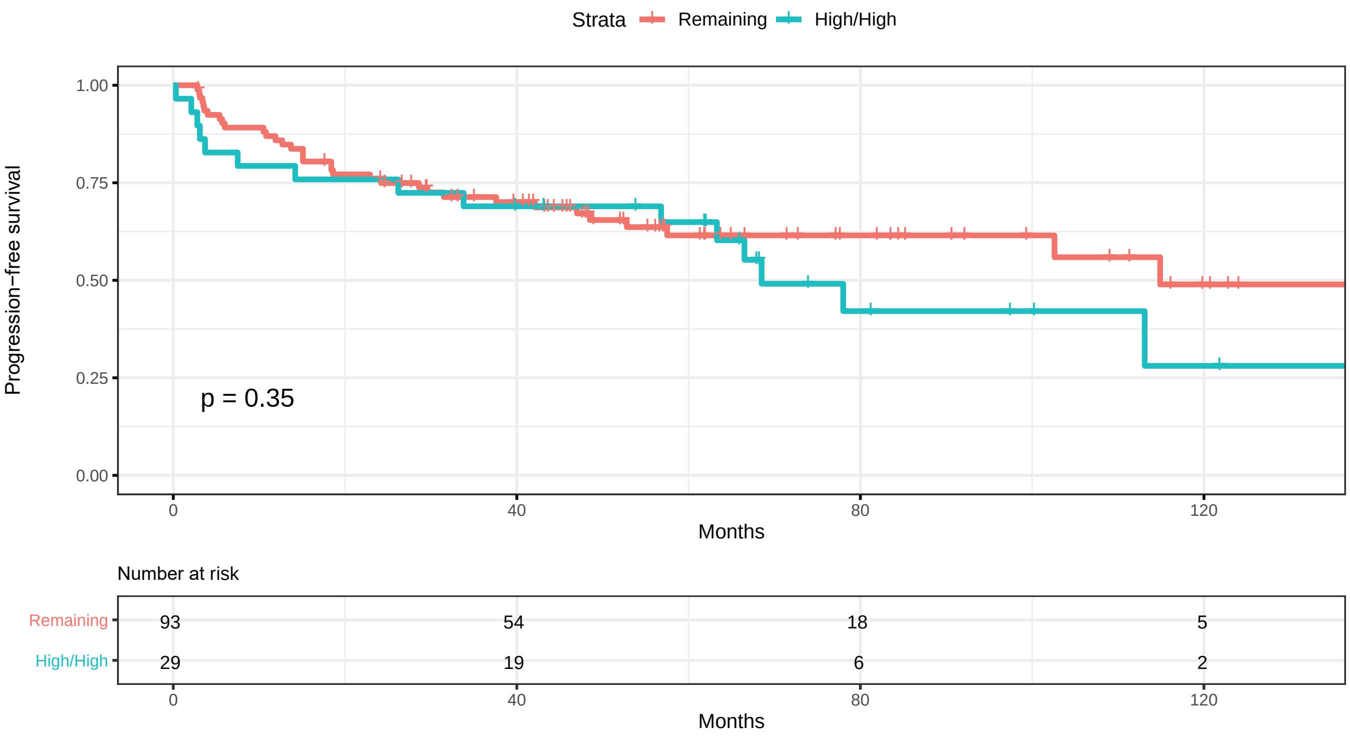

Figure s4. Progression free survival comparison between High/High and Remaining. Following radical prostatectomy, patients' PSA levels were continually monitored in the clinic. Recurrence was defined as PSA level >0.2 ng/mL. (+) Indicates patients without BCR censored at most recent PSA measurement. Kaplan-Meier analysis was conducted using RStudio with P-value showing log-rank test.4 patients were excluded (3 from group 1 and 1 from group 2, as PSA follow-up was not conducted)

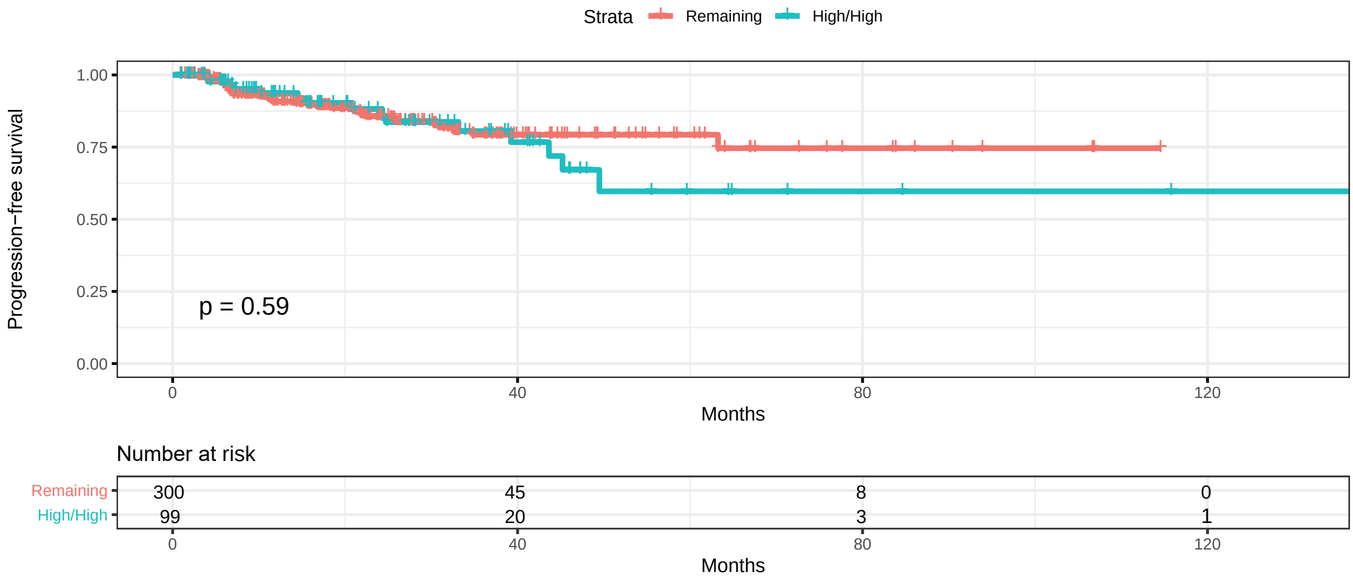

Figure s5. Progression free survival comparison between High/High and Remaining in the PRAD-TCGA cohort. Following radical prostatectomy, patients' PSA levels were continually monitored in the clinic. Recurrence was defined as PSA level >0.2 ng/mL. (+) Indicates patients without BCR censored at most recent PSA measurement. Kaplan-Meier analysis was conducted using RStudio with P-value showing log-rank test.

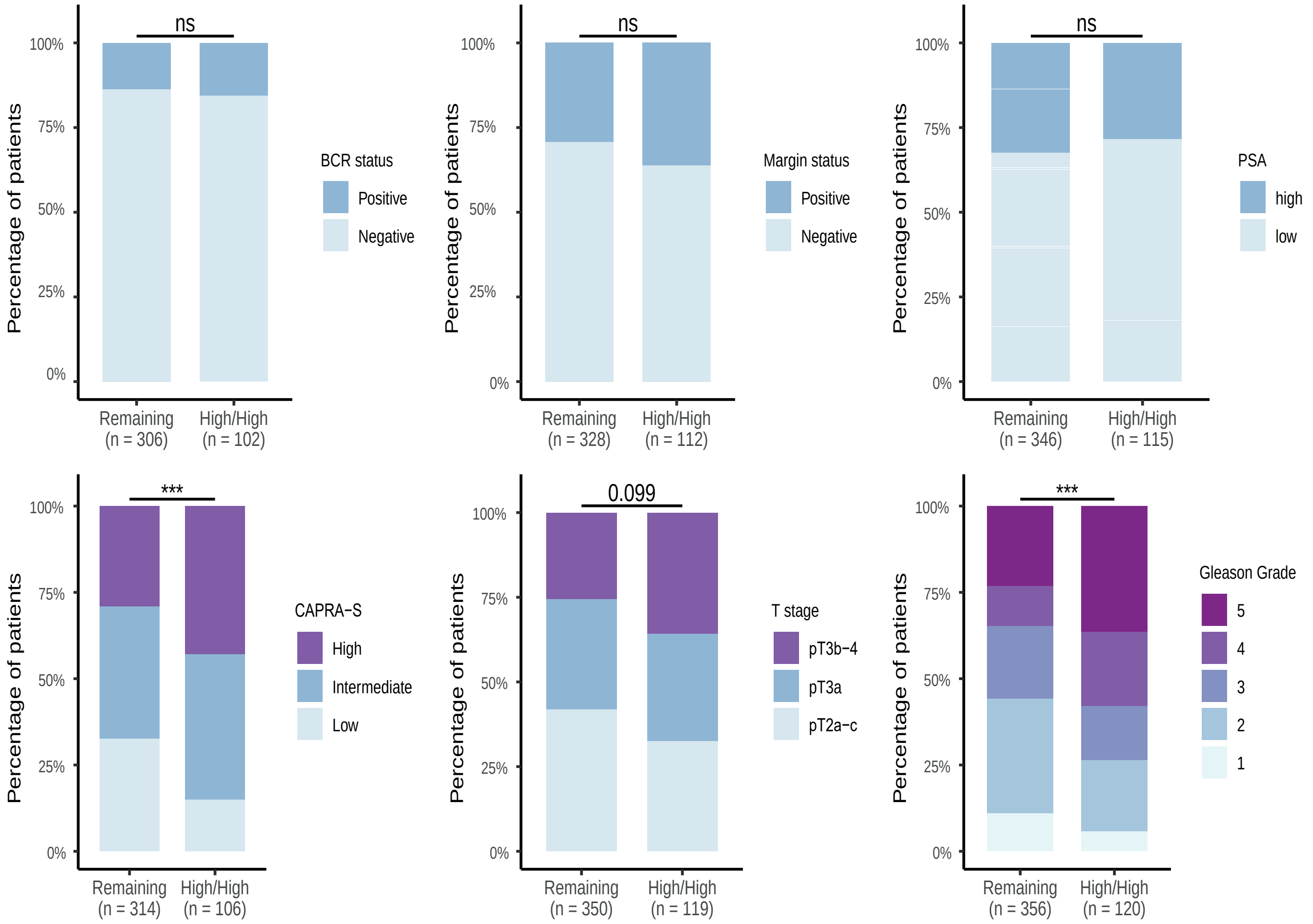

Figure s6. Prostate cancer characteristics in relation to PSMA expression and CD8$^+$ T-cell infiltration in LPC tissue samples from PRAD-TCGA samples. Patients were stratified (Remaining versus High/High) based on the median PSMA expression and median CD8$^+$ T-cell enrichment score, with patients scoring above median in both categories being placed into High/High. Statistical analysis was conducted in Rstudio (Fisher's exact test: *<0.05, **<0.01, **<0.001, ****<0.0001, ns = non-significant)

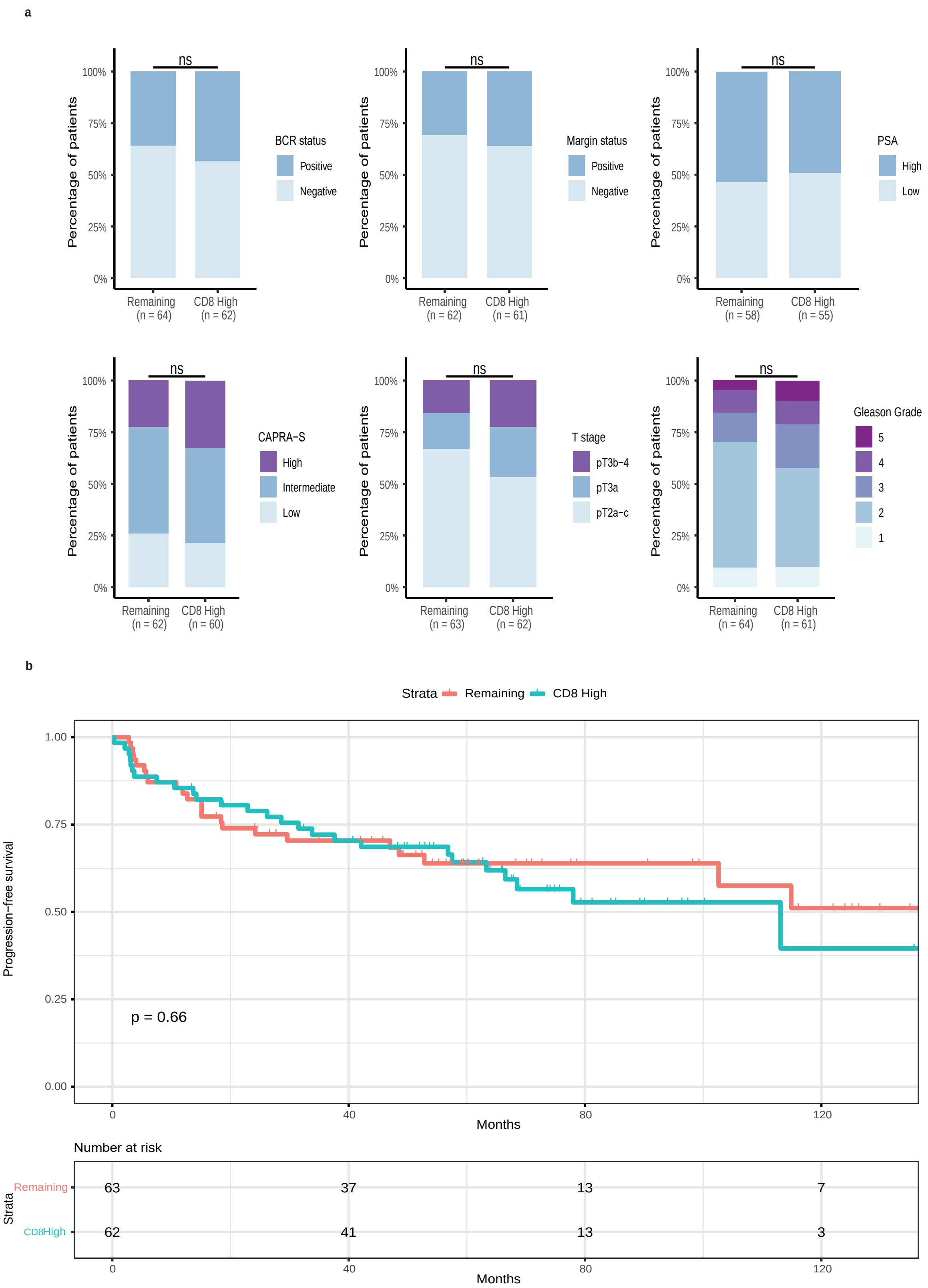

Figure s7. Prostate cancer characteristics **a** and Kaplan-Meier analysis **b** in relation to CD8^{+} T-cell infiltration in LPC tissue. Patients where stratified (Remaining versus CD8 high) based on median CD8^{+} T-cell enrichment score, with patients scoring above median being placed into CD8 High. Recurrence was defined as PSA level >0.2 ng/mL. (+) Indicates patients without BCR censored at most recent PSA measurement. Statistical analysis was conducted in Rstudio (Fisher's exact test: *<0.05, **<0.01, ***< 0.001, ****<0.0001, ns = non-significant.

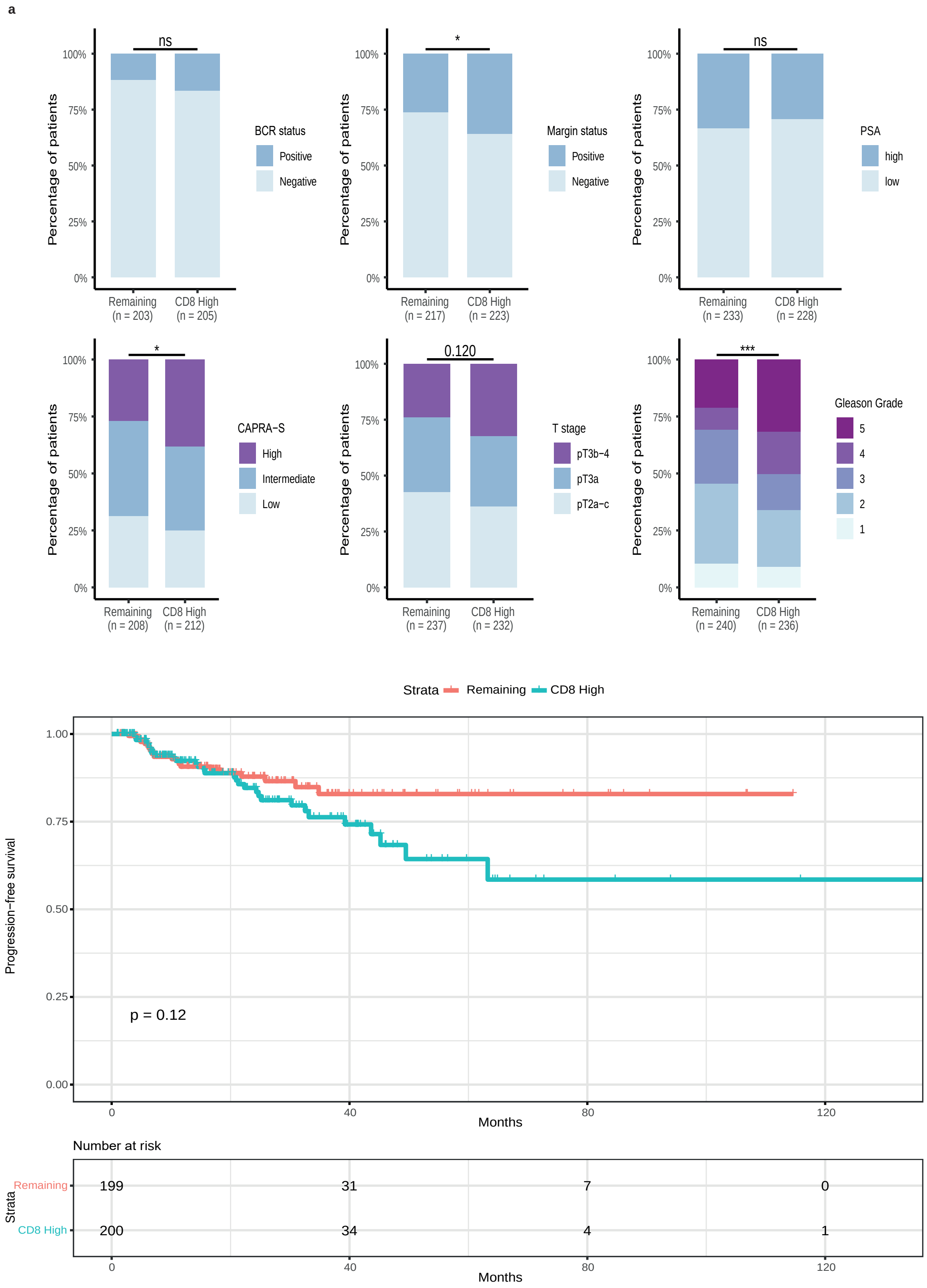

Figure s8. Prostate cancer characteristics **a** and Kaplan-Meier analysis **b** in relation to CD8$^+$ T-cell infiltration in LPC tissue. Patients where stratified (Remaining versus CD8 high) based on median CD8+ T-cell enrichment score, with patients scoring above median being placed into CD8 High. Recurrence was defined as PSA level >0.2 ng/mL. (+) Indicates patients without BCR censored at most recent PSA measurement. Statistical analysis was conducted in Rstudio (Fisher's exact test: *<0.05, **<0.01, ***< 0.001, ****<0.0001, ns = non-significant.

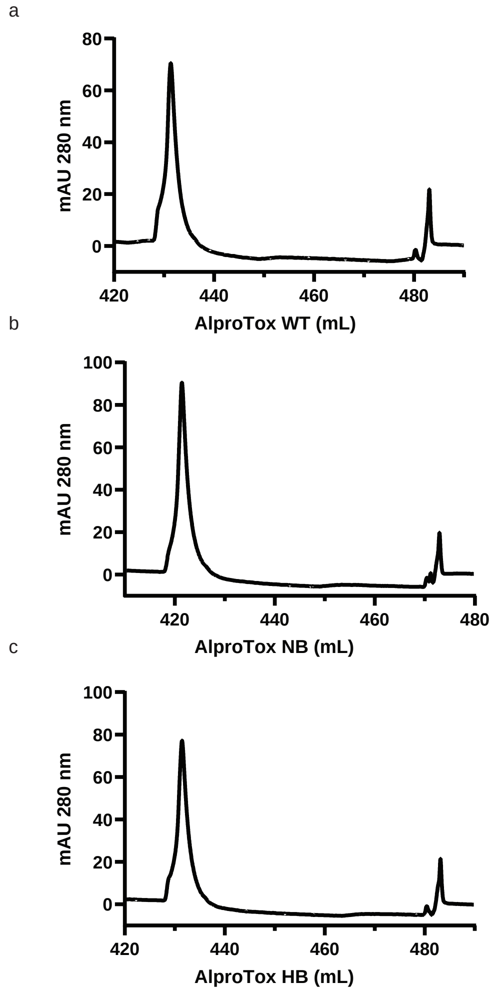

Figure s9. Chromatogram generated from ÄKTA^{TM} purification system of **a** AlproTox WT, **b** AlproTox NB and **c** AlproTox HB (c). Absorbance is measured on they y-axis with mL flowthrough and collected fractions displayed on x-axis. An anti-albumin CaptureSelect column was used for purification.

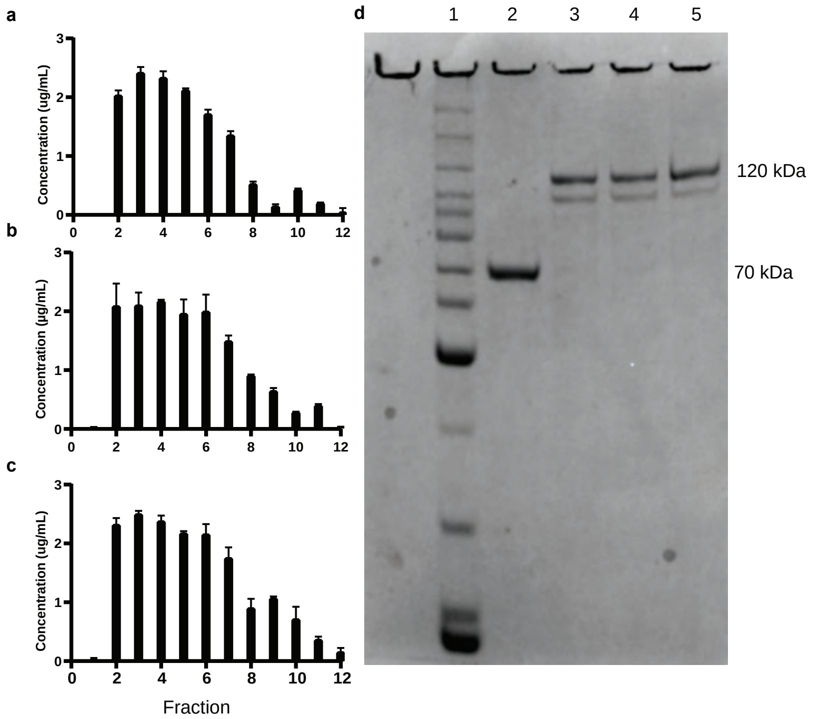

Figure s10. Bar plot depicting concentration of eluted fraction following ÄKTA^{TM} purification (**a** AlproTox WT, **b** AlproTox HB, **c** AlproTox NB) and SDS-PAGE following concentration of fractions visualized with Coomassie blue staining (**d**). ELISA was used to determine concentration using an anti-albumin antibody. **d** 10 % SDS. Lane 1. Ladder, lane 2. HSA, lane 3. AlproTox WT, lane 4.AlproTox HB, lane AlproTox NB. Statistical analysis was made in Graphpad prsim with mean ±SD shown from 1 ÄKTA^{TM} purification of each AlproTox variant with three technical replicates.

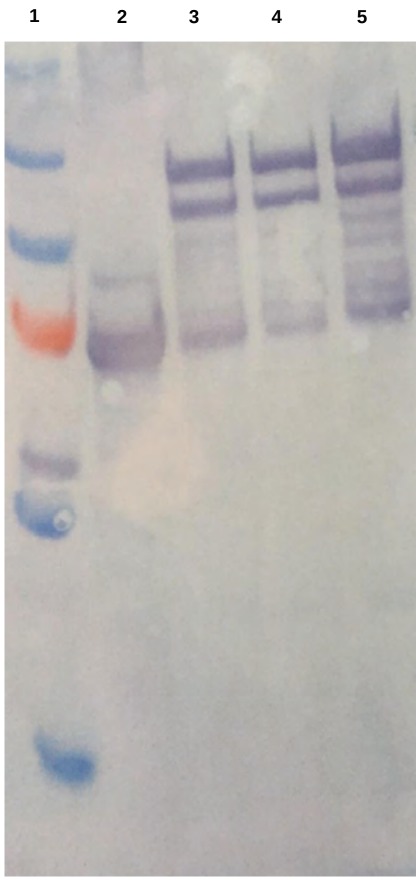

Figure s11. Western blot of AlproTox fusions. Lane 1. Ladder, lane 2. HSA, lane 3. AlproTox HB, lane 4. AlproTox WT, lane 5. AlproTox NB. An anti-albumin HRP-conjugated antibody was used for detection and gel was run together with pre-stained ladder
