# Supplementary material for: Identification of a high-risk immunogenic prostate cancer patient subset as candidates for T-cell engager immunotherapy and the introduction of a novel albumin-fused anti-CD3 × anti-PSMA bispecific design
Source: Br J Cancer. 2022 Oct 15;127(12):2186–97. doi: 10.1038/s41416-022-01994-1 (PMC9727128; doi:10.1038/s41416-022-01994-1)
Supplement: Supplementary file 3 — Supplementary methods [file 41416_2022_1994_MOESM3_ESM.docx]

**cDNA design and expression vectors**

The AlproTox cDNA construct was synthesised (Genscript Biotech, Piscataway, New Jersey, USA) with unique restriction sites between each protein domain. The bispecific antibody is composed of an anti-PSMA human domain antibody (sequence from patent WO 2017/122018 Al, Crescendo biologics, Cambridge, United Kingdom) and the anti-CD3 scFv clone; OKT3. The synthesised bispecific was subcloned into vectors containing wild-type HSA and variants with high FcRn binding affinity (HB) or removed FcRn binding affinity (NB).

**Protein expression and purification**

HEK293 E cells were transiently transfected using PEImax (Polysciences, Warrington, Pennsylvania, USA #24765-1) to produce the HSA fusions. Following transfection, cells were cultivated in serum-free Freestyle 293 Medium (Gibco, #12338-018). Secreted protein was purified from the supernatant by using a CaptureSelect HSA affinity matrix (Thermo Fisher Scientific, Waltham, Massachusetts, USA #191297005) mounted on an ÄKTA^TM^ start protein purification system. Protein was eluted by a 2 M MgCl_2_ PBS (pH 7,4) buffer and decanted to Vivaspin® 2 50.000 MWCO columns (Satorius AG, Göttingen, Germany) with buffer exchanged into PBS.

**Polyacrylamide gel electrophoresis and western blot analysis**

Samples were heated and run on 10 % SDS PAGE with 500 ng protein loaded and visualised by Coomassie brilliant blue or alternatively blotted onto a PVDF membrane and blocked in 2 % skim milk solution in PBS. Bands were visualised using an anti-albumin HRP conjugated antibody (Sigma-Aldrich, #A-7544) diluted 1:2500 in mPBS, washed three times and developed using on membrane TMB (Sigma, #T0565).
